# Supplementary material for: Regional adaptations and parallel mutations in Feline panleukopenia virus strains from China revealed by nearly-full length genome analysis
Source: PLoS One. 2020 Jan 16;15(1):e0227705. doi: 10.1371/journal.pone.0227705 (PMC6964837; doi:10.1371/journal.pone.0227705)

Supplementary material;

Regional adaptations and parallel mutations in Feline panleukopenia virus strains from China revealed by nearly-full length genome analysis

Élcio Leal [1,2,*,†], Ruiying Liang [1,3,†], Qi Liu[1], Fabiola Villanova[2],Lijun Shi[1,3], Lin Liang[1,3], Jinxiang Li[1,*], Steven S. Witkin[4], Shangjin Cui[1,3,*]
[1]Institute of Animal Sciences, Chinese Academy of Agricultural Sciences, Beijing 100193, China; (Q.L) qiliu0223@163.com; (L.L.) liang-lianglin@caas.cn; (J.L.) Li-jinxiang@caas.cn; (S.C.) cuishangjin@caas.cn

[2]Federal University of Pará, Belém, Pará, 6075-000 Brazil; (E.L) elcioleal@gmail.com; (F.V.) fvillanova@gmail.com

[3]Beijing Observation Station for Veterinary Drug and Veterinary Biotechnology, Ministry of Agriculture, Beijing 100193 China; (R. L) ruiyingliang89@163.com ; (L.S) shilijun@caas.cn

[4]Department of Obstetrics and Gynecology, Weill Cornell Medicine, New York, NY 10065 USA and Institute of Tropical Medicine, Sao Paulo, Brazil; (S.S.W.) switkin@med.cornell.edu

* Correspondence: lijinxiang@caas.cn (Jinxiang Li), cuishangjin@caas.cn (Shangjin Cui), elcioleal@gmail.com (Elcio Leal)

2 supplementary material; Figure 1A.

FPLV colloidal gold test strip. A) negative control. B) positive sample

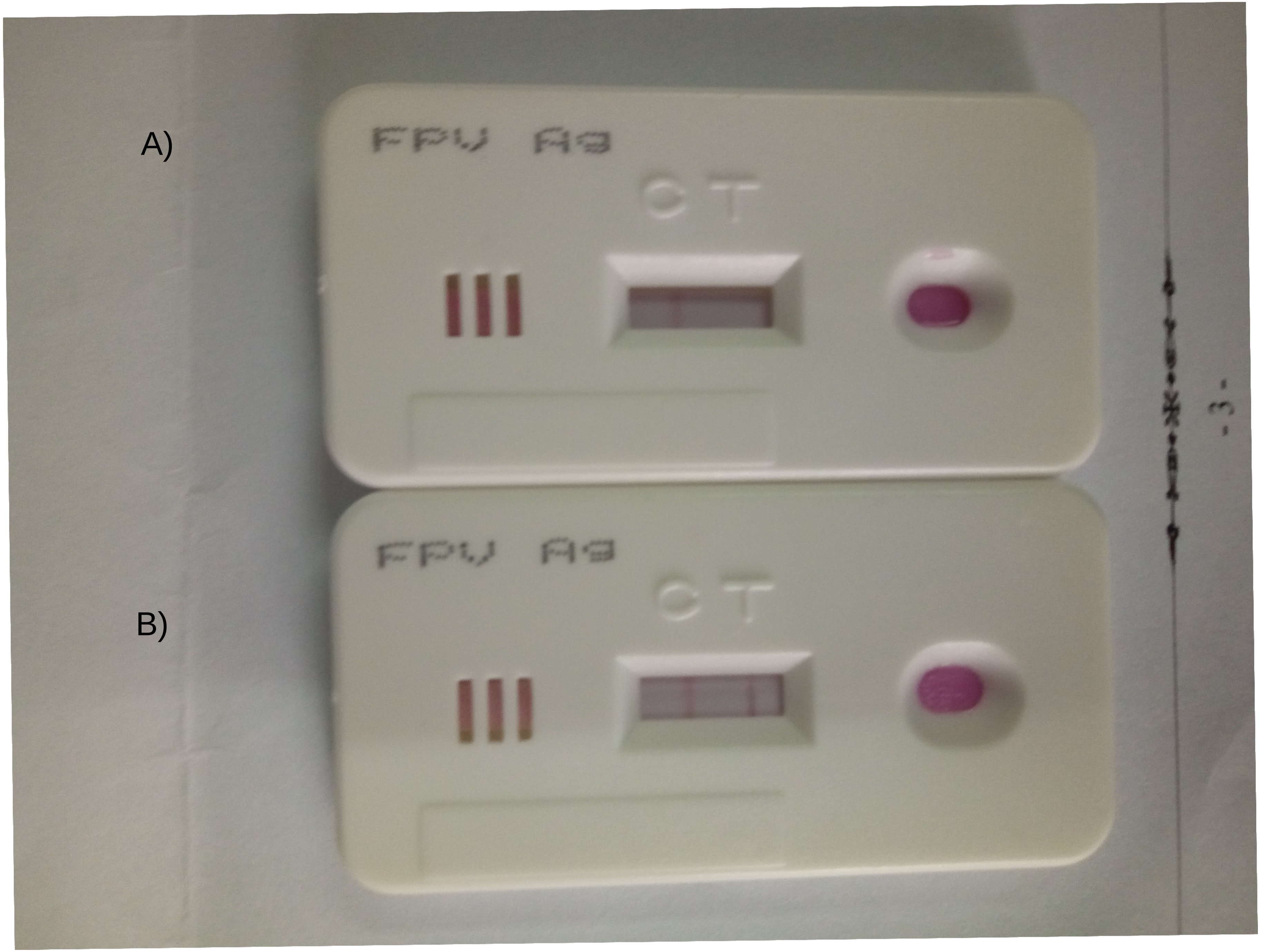

3 supplementary material; Figure 1B. Agarose gel of PCR

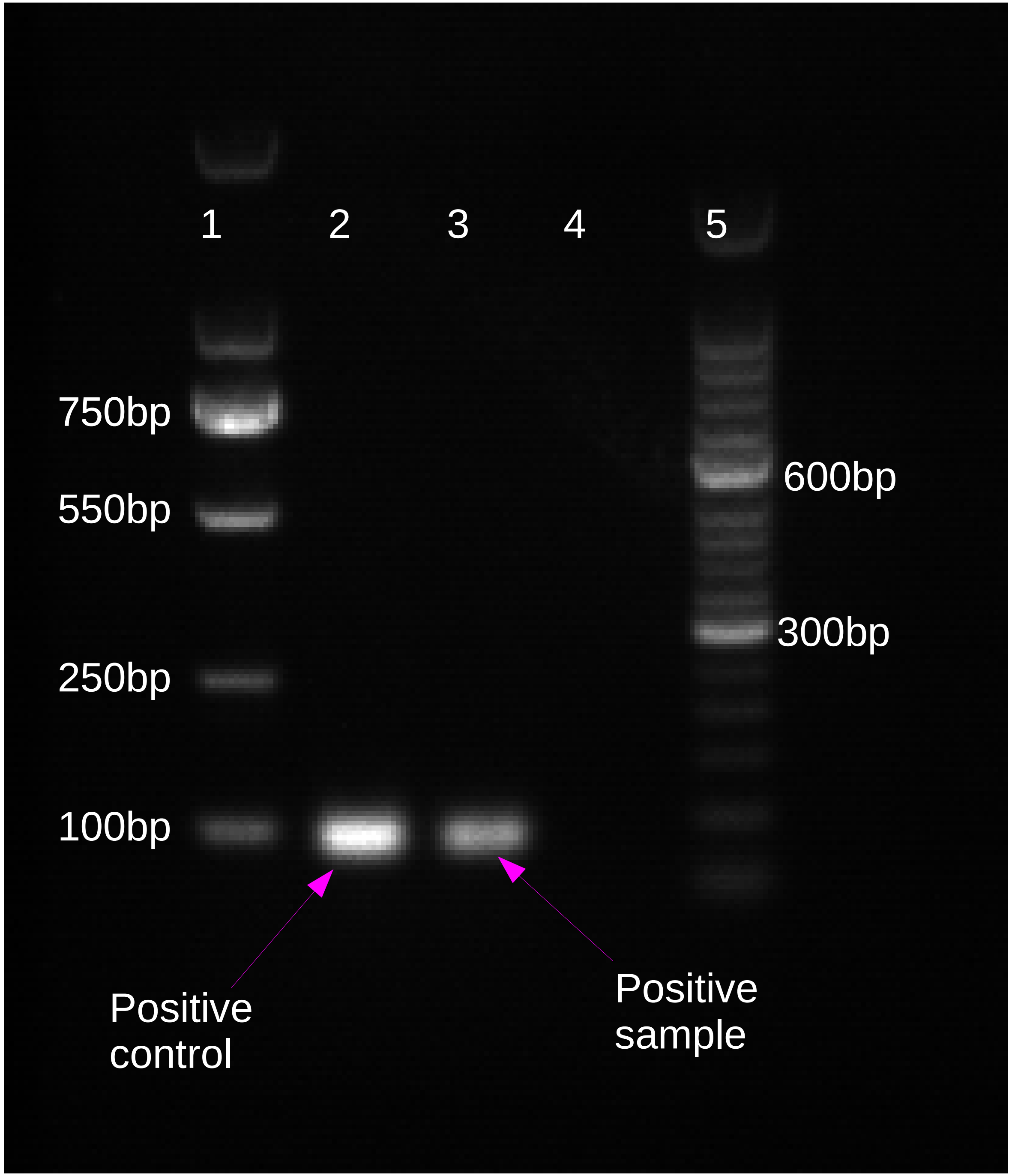

4 supplementary material; Figure 2A.
cytopathic effect induced by FPLV in CRFK cells
visualized by inverted microscopy at magnification of 200 µm 72 hours post infection.

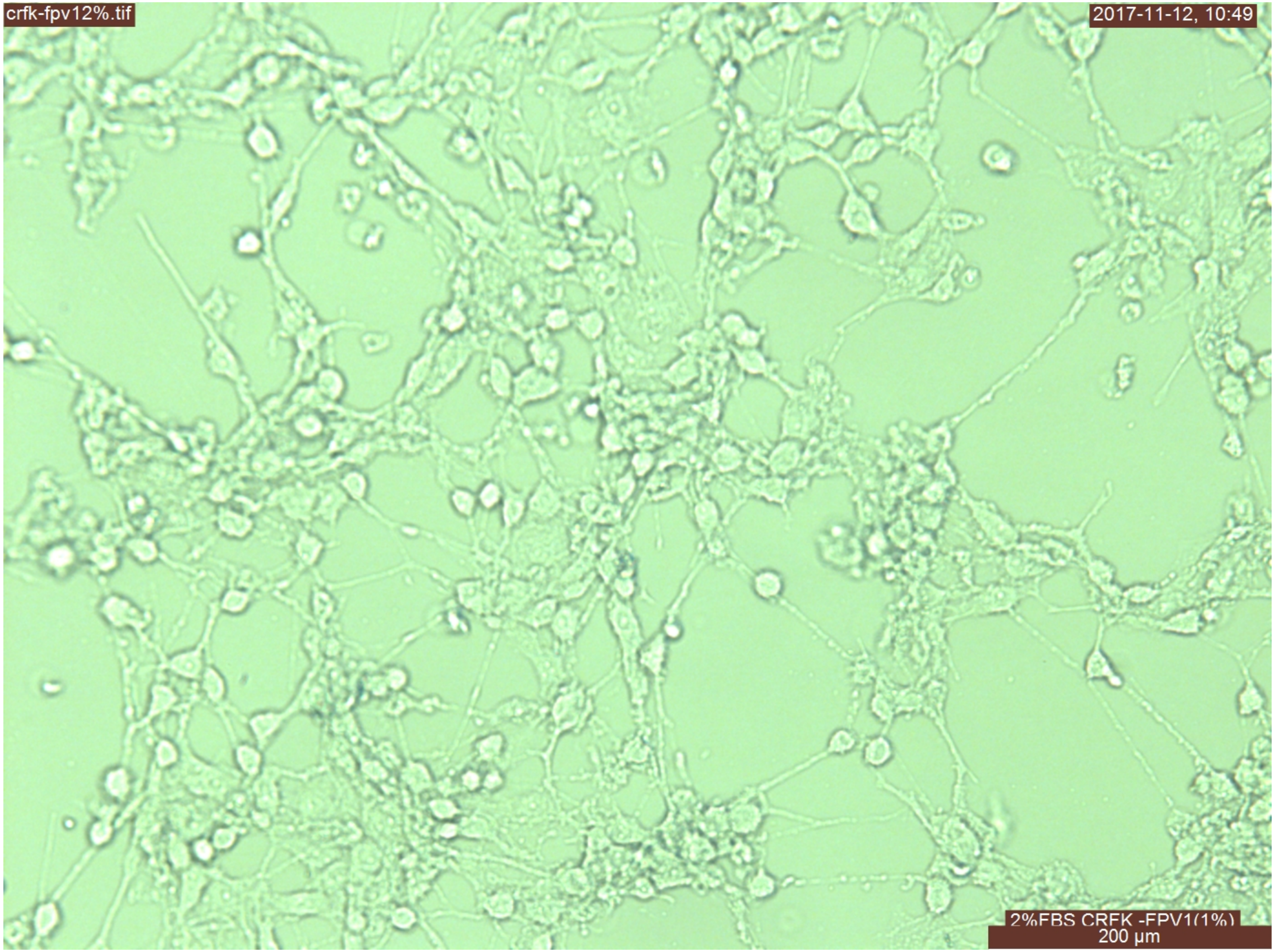

5 supplementary material; Figure 2B negative control of uninfected CRFK cells.

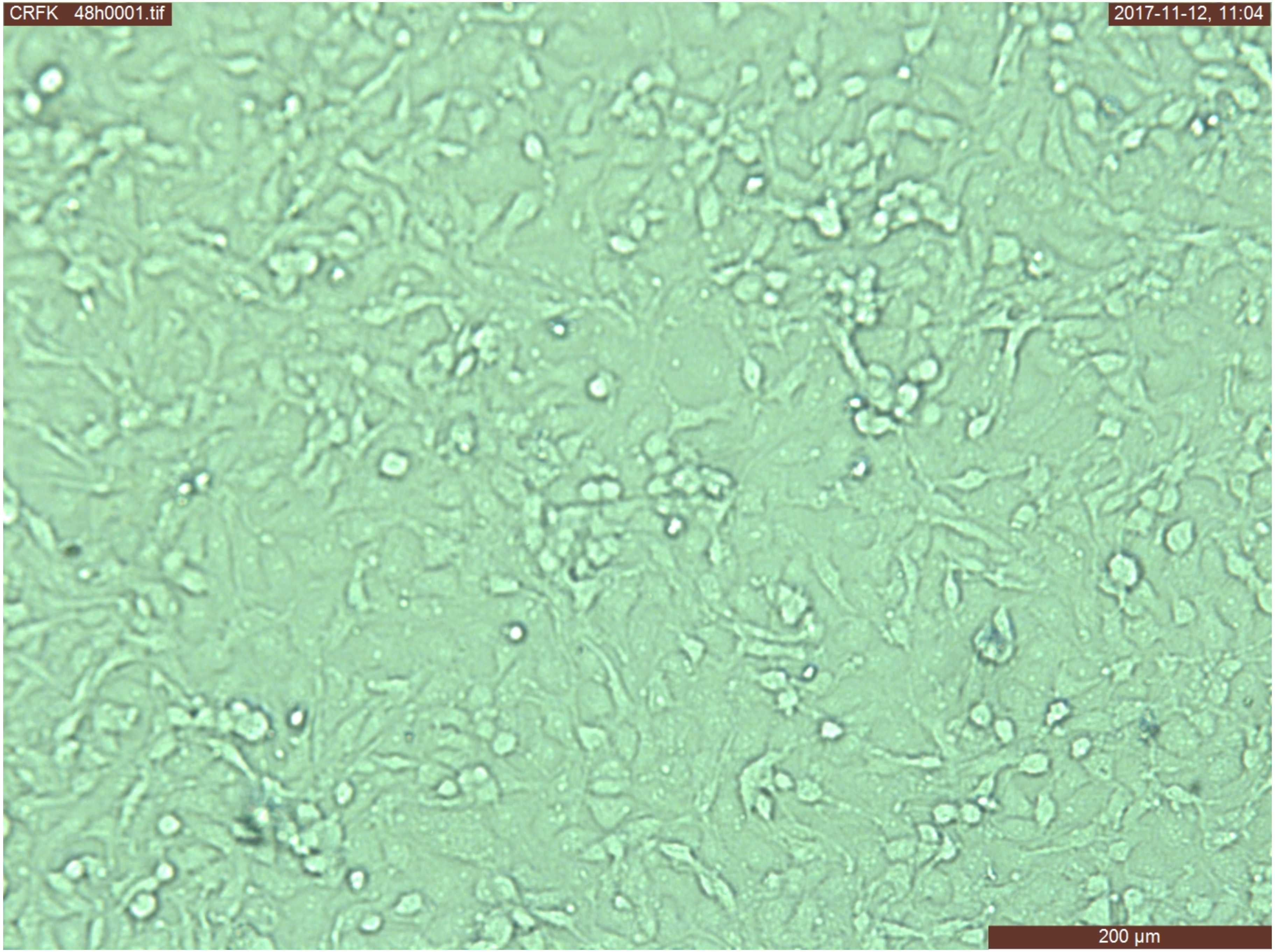

6 supplementary material;

Figure 3A. Immunofluorescence staining of CRFK cells after infection with FPLV. A green color indicates positive staining of FPLV (200×)

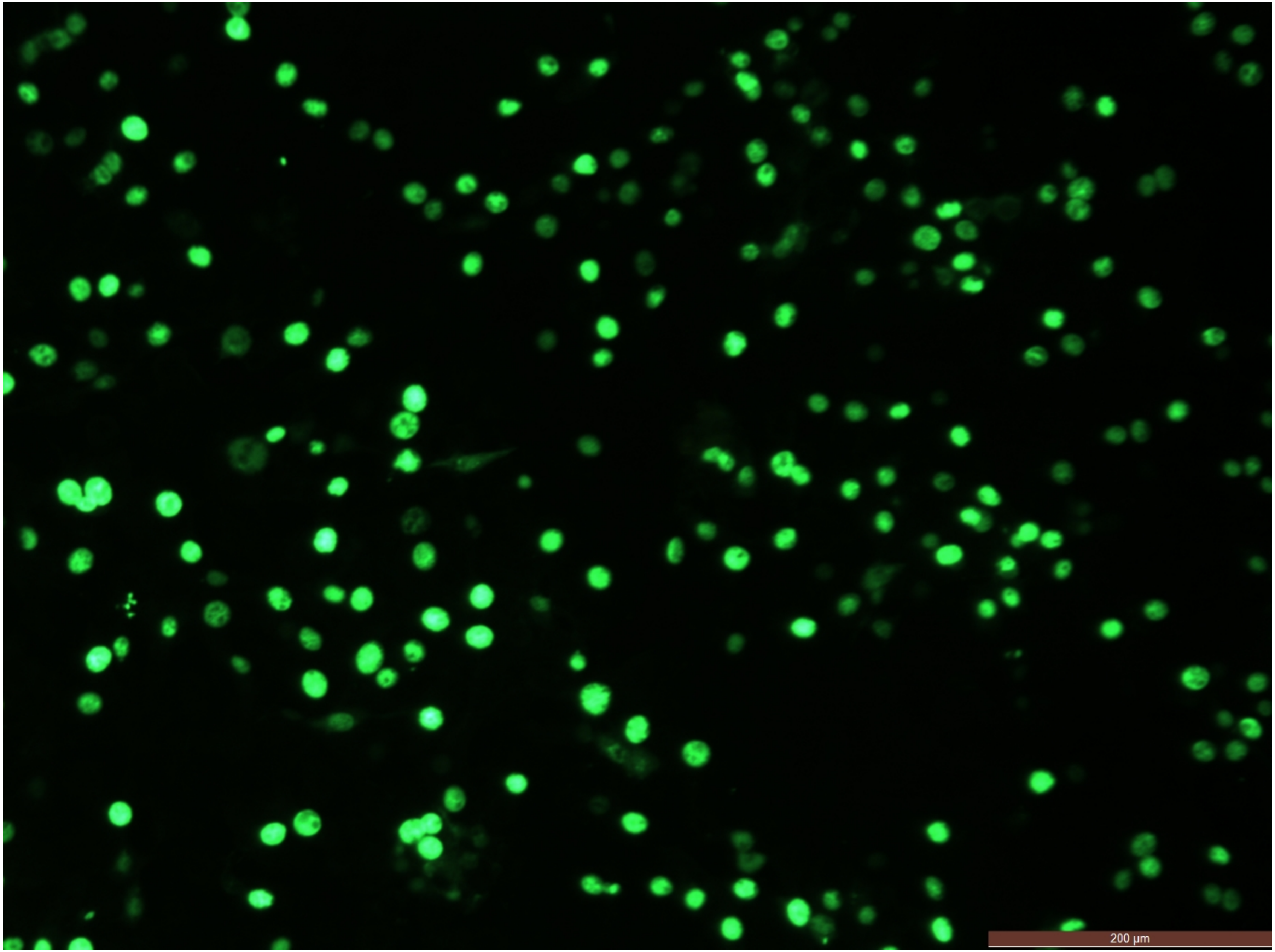

7 Supplementary material; Figure 3B.

Uninfected CRFK cells served as a negative control

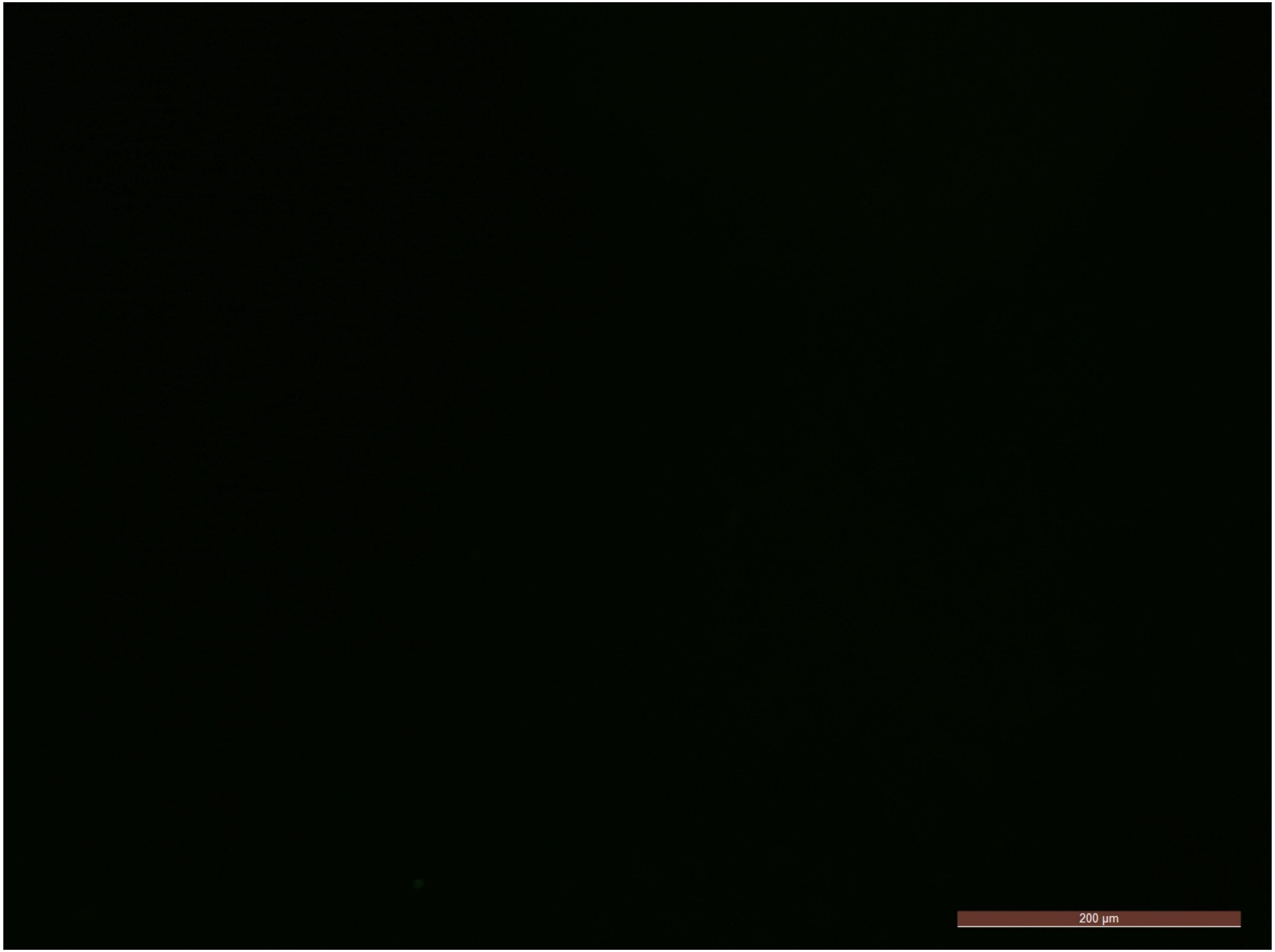

Supplement: S1 Raw Images — (PDF) [file pone.0227705.s001.pdf]
