# Supplementary material for: Regional adaptations and parallel mutations in Feline panleukopenia virus strains from China revealed by nearly-full length genome analysis
Source: PLoS One. 2020 Jan 16;15(1):e0227705. doi: 10.1371/journal.pone.0227705 (PMC6964837; doi:10.1371/journal.pone.0227705)
Supplement: S1 Table — (PDF) [file pone.0227705.s002.pdf]

Table S1

Identification of FLPV sequences used in the phylogenetic analyses

GenBankID	SequenceName
MH165481	BJ04
MH165482	BJ05
EU698028	Tai`an
M10824	FPV
D00765	MEV/Abashiri
U22190	MEV/d
JX475253	CO/546/10
EU498701	42/06
AY665657	MEV/Rodniki
AY665656	MEV/Beregovoj
GU392247	MEV/Shangdong3
FJ592174	MEV/China
EU498707	42/06
FJ440713	ARG07
EU018144	ARG02
FJ440711	ARG05
FJ440712	ARG06
EU018143	ARG03
EU018145	ARG01
EU018142	ARG04
FJ440714	ARG08
KJ813893	FPV/Bobcat/ND/979/2013
U22189	FPV
JX475259	CO/545/10
JX475254	CO/977/10
JX475245	CO/952/10
EU498720	498/07
EU498718	443/07
AF015223	Taiwan
M23999	MEV
D78584	TU10
EU659114	FPV
EU659113	FPV
EU498713	97/06
D88286	FPV
MF069446	Raccoon/RC9/BC
KM624023	FPV/Raccoon/TX/Rac3/1978
JN867595	FPV/Raccoon/TX/Rac2.2/78
JN867593	FPV/Raccoon/CA/208
JN867596	FPV/Raccoon/TX/Rac1.2/78
MF069447	Raccoon/RC18/BC
MF069445	Raccoon/RC6/BC
JN867594	FPV/Raccoon/NJ/RPV
KJ813895	FPV/Raccoon/MA/188/2012
KJ813894	FPV/Raccoon/MA/190/2012
AB000054	Fukagawa
AB000070	TU8
M24004	CU4
EU659111	FVP
U22191	MEV/e
HQ184197	KS11
HQ184193	K22
EU252145	KF001c

cont.

HQ184192	K7
EU498717	50/07
X55115	FPV
EU498714	97/06
U22187	FPV
EU498680	Purevax
M24002	strainPhilipsRoxane
D88287	PLI
JX475270	GA/1/12
EU498719	490/07
EU498681	Felocell
FJ712220	MEV/MDQ
FJ712221	MEV/LYT
GU392246	MEV/Shangdong5
GU392245	MEV/Shangdong4
GU392249	MEV/Shangdong1
GU392248	MEV/Shangdong2
GU392255	MEV/Shangdong1
FJ712218	MEV/Dalian
GU392254	MEV/Dalian1
GU392251	MEV/Dalian1
GU272029	MEV/ZYL
FJ712217	MEV/Suning
EU137663	MEV/China
GU392253	MEV/Jilin1
HM015824	MEV/DL
EF428258.1	Mink
GU392258	MEV/Dalian2
KT899745	MEV/LHV
KP008112	MEV/China
HQ883275	MEV/Jilin/2010
KY094119	MEV/SD8
KY094118	MEV/SD7
FJ712219	MEV/Manzhouli
GU392252	MEV/Dalian2
GU392250	MEV/Dalian2
HQ694567	MEV/LN
GU272028	MEV/ZYL
U22188	FPV
JX475256	CO/1103/11
EU360959	1335/07
KP280068	HRB
FJ405225	FPV
DQ474235	HT
EU498688	134/04
EU498695	119/05
EU498685	150/03
EU498711	42/06
EU498712	42/06
EU498694	20/05
EU498684	103/02
EU498709	42/06
EU498700	42/06
KT240131	PT022/08
EU221280	cat/39897/PT06
EF418569	Lion/PT06
EF418568	Tiger/PT06

cont.

EU221279	cat/46912/PT05
EU221278	cat/52171/PT05
EU498715	228/06
EU498691	134/04
EU498708	42/06
EU498702	42/06
EU498696	22/06
EU498710	42/06
EU498703	42/06
EU498689	134/04
EU498697	42/06
EU498698	42/06
DQ474236	JF
KX900570	HH
KX943318	IZSSI
EU498693	355/04
EU659115	kai
HQ184196	K50
KX685354	HN
EU360958	933/07
EU498682	198/01
DQ474238	SM
EU498706	42/06
EU252147	KF003
HQ184195	K49
EU498699	42/06
EU252146	KF002
EF988660	XJ
FJ936171	ChangC2007
EU498704	42/06
EU498705	42/06
EU498692	143/04
EU498686	189/03
KY094115	MEV/SD4
KY094113	MEV/SD2
KY094117	MEV/SD6
KY094116	MEV/SD5
KY094114	MEV/SD3
